# Population awareness of cardiovascular disease and its risk factors in Buea, Cameroon

**DOI:** 10.1186/s12889-017-4477-3

**Published:** 2017-06-05

**Authors:** Leopold Ndemnge Aminde, Noah Takah, Calypse Ngwasiri, Jean Jacques Noubiap, Maxime Tindong, Anastase Dzudie, J. Lennert Veerman

**Affiliations:** 1 School of Public Health, Faculty of Medicine, The University of Queensland, QLD, Brisbane, 4006 Australia; 2 Non-communicable diseases Unit, Clinical Research Education, Networking and Consultancy (CRENC), Douala, Cameroon; 3 Department of Internal Medicine, Faculty of Health Sciences, University of Buea and Douala General Hospital, Douala, Cameroon; 4 London School of Hygiene and Tropical Medicine, London, UK; 5 Department of Medicine, Groote Schuur Hospital and University of Cape Town, Cape Town, South Africa; 6 Universite Libres de Bruxelles, Brussels, Belgium; 7 NIH Millennium Fogarty Chronic Disease Leadership Program and Soweto Research Group, Department of Medicine, University of Witwatersrand, Johannesburg, South Africa; 8 Cancer Research Division, Cancer Council New South Wales, Sydney, NSW Australia

**Keywords:** Cardiovascular disease, Awareness, Risk factors, Stroke, Heart attack, Cameroon

## Abstract

**Background:**

Adequate awareness of cardiovascular diseases (CVD) and their risk factors may help reduce the population’s exposure to modifiable risk factors and thereby contribute to prevention and control strategies. There is limited data on knowledge among the general population in sub-Saharan Africa regarding CVD and risk factors. We aimed to assess the population awareness (and associated factors) of CVD types and risk factors in Buea, Cameroon.

**Methods:**

This was a community-based cross-sectional study conducted in 2016 among randomly selected adults (>18 years). Data on socio-demographic characteristics, knowledge about CVD types, their risk factors and warning signs for CVD events (stroke and heart attack) were acquired using a self-administered questionnaire. Logistic regression analysis was used to investigate factors associated with moderate-to-good knowledge.

**Results:**

Of the 1162 participants (61.7% women, mean age 32 years), 52.5% had overall poor knowledge (mean score 12.1 on total of 25) on CVD with only about a quarter correctly identifying types of CVD. Overall, 36, 63 and 45% were unaware of CVD risk factors, warning signs of heart attack and stroke respectively. In multivariable analysis; high level of education (aOR = 2.26 (1.69–3.02), *p* < 0.0001), high monthly income (aOR = 1.64 (1.07–2.51), *p* = 0.023), having a family history of CVD (aOR = 1.59 (1.21–2.09), *p* = 0.001) and being a former smoker (aOR = 1.11 (1.02–1.95), *p* = 0.043) were associated with moderate-to-good knowledge.

**Conclusions:**

There exists a significant gap in population awareness about CVDs in Cameroon and this is similar to previous reports. Cost-effective community health education interventions taking into account socioeconomic status may be beneficial in this setting.

## Background

Non-communicable diseases (NCD) account for over 1.4 billion disability adjusted life years (DALYs), which is 58.5% of the global health burden [1]. Cardiovascular diseases (CVD) are a major contributor to this global NCD burden and the leading cause of deaths around the world [2]. In 2012, the World Health Organization (WHO) estimated that over 17 million people died from CVDs, representing over a third of global mortality. The majority of these CVD deaths were due to ischaemic heart disease (7.4 million) and stroke (6.7 million). Low and middle income countries (LMIC) are unduly hit by this CVD epidemic with three-quarters of these premature CVD deaths occurring there [3]. According to estimates based on the largest African acute heart failure registry, almost 1 in 5 persons die from acute heart failure within 6 months of diagnosis [4]. This CVD epidemic is largely driven by behavioural and environmental risk factors whose magnitude seems to rapidly increase in SSA owing to epidemiologic transition and population ageing [5]. CVD events (mostly stroke and heart failure) are the main endpoint of most CVDs and feature among the five leading causes of DALYs from NCD [1]. Awareness of chronic diseases including CVDs and their risk factors can be a precondition for success in prevention and control of these diseases [6, 7]. This knowledge will inform individuals on healthy attitudes to adopt [8, 9] and to be proactive in reducing their own lifetime risk by plummeting their exposure to modifiable risk factors for CVD [10, 11]. Studies have assessed the knowledge of adult populations regarding CVD [12–15], and even among school children [16], however studies from Africa are scant and concentrate on specific populations such as primary care physicians in western Cameroon [17], slum dwellers in Nairobi [18], and university staff and students in Nigeria [19]. Studies around CVD and its risk factors in sub-Saharan Africa have similarly shown some variation in the distribution/affection across genders [20]. It is not known whether this difference extends to their knowledge levels regarding these diseases in Cameroon. There are limited reports from Cameroon in the general population, and studies have shown that the development of targeted community health education and promotion programs may require assessment of population knowledge about these CVDs [10]. Hence, we sought in this study to estimate current levels of awareness regarding types of CVDs, their risk factors and warning features for CVD events (stroke and heart attack), as well as explore the knowledge differential by sex in the adult population of Buea, Cameroon.

## Methods

### Study design, setting, population and sampling

This was community-based cross-sectional study conducted during 1 April to 30 May, 2016 in the city of Buea. This town is the administrative capital of the Southwest region of Cameroon and covers a total surface area of 870 km^2^. It has a population of about 200,000 persons [21]. It has a regional hospital (secondary care level) which serves as a referral hospital in the southwest region. The Buea health district (BHD) is divided into seven health areas (Bokwango, Buea Town, Bova, Buea Road, Molyko, Muea and Tole). The target population for this study was all adults (≥ 18 years) residing in Buea during the study period.

The minimum sample size was determined using an online sample size calculator [22]. Considering a population of 200,000 persons, with a confidence level of 95% and a 0.03 margin of error, this gave an estimated minimum sample size of 1061. A total of 1162 adult participants were finally recruited for the study. In order to get a representative study sample, a multistage sampling technique was then used to recruit eligible participants for the study. In each of the 7 health areas in the BHD, a systematic random sampling was used whereby a bottle was spun [23, 24], and the direction of the bottle was used to choose the first house to be surveyed. Thereafter, every 4th house was considered. Via simple random sampling, balloting of eligible members in each household was done and only one participant was chosen.

### Study procedure and ethical issues

The study was approved by the Ethics committee of the Southwest Regional delegation of the Ministry of Health (MOH) of Cameroon. Participants were explained the components of the study and only those who freely consented were included. Confidentiality was maintained. A structured self-administered questionnaire in English language was adapted from previous validated published studies [10, 15, 25] and also guided by WHO CVD fact sheet [3]. The content validity of the questionnaire was established through consultation within the working group and with experts. The questionnaire had three sections: socio-demographic characteristics; personal health assessment (e.g. perception of health and weight status, frequency of physical activity per week, perception of overall lifestyle, consumption of healthy food (rich in fruits and vegetables, high fibre and low in saturated fat and salt respectively), alcohol use and smoking, family history of CVD); assessment of knowledge about CVDs, their risk factors and symptoms of stroke and heart attack. Questions regarding diet were defined according to WHO recommendations for healthy diet, e.g. a diet rich in fruit and vegetables was defined as an individual having at least five servings of fruits and vegetables per day; low salt diet was considered as not consuming more than a tea spoon of salt per day [26]. Smoking status was defined as never smoker, former smoker (an individual who has smoked in the past but reported not smoking at the time of the study) and current smoker (an individual who confirmed to be smoking at the time of the study). Alcohol use was simply evaluated as the consumption of greater than 10 (5 for women) local beers per week (1 local beer contains 28 g of alcohol) [27]. Physical activity was defined following WHO recommendations for physical activity in adults, as the number of times in a week an individual did at least 30 mins of moderate intensity exercise (such as running, cycling, walking, and jogging) per day [28]. The questionnaire was first pre-tested on 5% (53 subjects) of estimated study sample and necessary modifications made thereafter to ensure the questionnaire was accurate and easy to comprehend. After completing the questionnaire, participants’ physical measurements were done using standard procedures (using a SECA scale for weight and stadiometer for height) with participants in light clothing and barefoot. The weight (kg) was measured to one decimal place. The height was measured to the nearest 0.5 cm using a portable stadiometer against the wall, and participants standing upright on flat surface without shoes with the back of the heels and occiput on the stadiometer. Body mass index (BMI) was calculated using the height and weight with the following formula: *weight [kg] /height [m]* ^*2*^ *.*

### Scoring of knowledge of participants

The questionnaire contained nine questions regarding knowledge of CVD risk factors, five questions each regarding knowledge on warning features of cerebrovascular disease and heart attack, and six questions regarding types of CVDs, giving a total of 25 questions. Each of these questions was equally scored (one point for each correct answer and zero otherwise). These points were then summed across all the questions. We categorised participants who obtained 20 or more correct responses having “good knowledge”, those with a score between 13 and 19 were classified as having “moderate knowledge” while those with a score of 12 and below were classified as having “poor knowledge”. Previous published studies [25] have used this categorization approach and hence assisted comparability of our study findings.

### Statistical analysis

Data were entered and analysed using IBM-Statistical Package for Social Sciences statistical software v.20 for Windows (SPSS Inc., Chicago, IL). Using descriptive statistics, we have summarized categorical variables as frequencies with proportions and continuous variables as means with standard deviations. Group comparisons were done using chi square test and independent samples t-test (or ANOVA) where appropriate. Overall knowledge score on CVD was dichotomized as low (score < 13 / 25) and moderate-to-good (score ≥ 13/25) knowledge. Bivariate logistic regressions were used to investigate factors associated with overall moderate-to-good knowledge for CVD. The following explanatory variables were included in the model: age, sex, level of education, income level, frequency of physical activity and healthy eating, family history of CVD, smoking status and alcohol use. Variables with *p* value ≤0.25 were included in the multivariable logistic regression model and adjusted for age and sex, to identify those with independent associations with moderate-to-good knowledge on CVD. A *p* value <0.05 was considered statistically significant.

## Results

### General characteristics

In all, of the 1250 adults invited to the study, 1162 (women = 717, 61.7%) participated in this community based study (response rate = 92.9%). The overall mean age was 31.5 years and was similar for both sexes (Table 1). Males had attained higher levels of education comparatively (*p* = 0.002). Overall, most participants had a monthly income of less than 50,000FCFA (~USD100), especially females (*p* < 0.0001). Thirty-two percent reported a family history of cardiovascular disease (CVD). Mean BMI was 25.2 kg/m^2^ with women being heavier (25.6 vs. 24.4 kg/m^2^; *p* < 0.0001), and most (85.9%) of the participants perceived their weight as being ‘normal’. Eleven and 21 % of the population reported smoking (current/former) and alcohol use respectively. Both characteristics were more frequent in men (both *p* < 0.0001). With respect to eating healthy food (diets rich in fruits and vegetables, low in saturated fats and low salt content), only 16.7% of the population reported doing so daily, while half of the participants reported doing 30 mins of physical activity per day on 3 or more occasions in a week (mostly the men; 57.9% vs. 47.9%, *p* = 0.001).

**Table 1:** General characteristics of the study population of adults in Buea, Cameroon, 2016

Characteristics	Male (*n* = 445, 38.3%)	Female (*n* = 717, 61.7%)	Total (*N* = 1162)	*p*-value
Age, mean ± SD	31.4 ± 12.8	31.6 ± 12.1	31.5 ± 12.4	0.747
Age category				0.032
18–24 years	191 (43.0)	268 (37.4)	459 (39.5)	
25–34 years	111 (25.0)	218 (30.4)	329 (28.3)	
35–44 years	64 (14.4)	108 (15.1)	172 (14.8)	
45–54 years	56 (12.6)	80 (11.2)	136 (11.7)	
55–64 years	12 (2.7)	36 (5.0)	48 (4.1)	
≥ 65 years	10 (2.3)	07 (1.0)	17 (1.5)	
Marital Status				<0.0001
Single	345 (77.7)	440 (61.5)	785 (67.7)	
Divorced	11 (2.5)	29 (4.1)	40 (3.5)	
Widowed	11 (2.5)	33 (4.6)	44 (3.8)	
Married	78 (17.4)	213 (29.8)	290 (25.0)	
Level of education				0.002
Up to secondary	90 (20.3)	209 (29.4)	299 (25.9)	
High school	102 (23.0)	138 (19.4)	240 (20.8)	
Diploma	41 (9.3)	86 (12.1)	127 (11.0)	
Undergrad University	173 (39.1)	231 (32.5)	404 (35.0)	
Postgrad University	37 (8.4)	47 (6.6)	84 (7.3)	
Employment status				<0.0001
Unemployed	51 (11.7)	86 (12.0)	137 (11.9)	
Retired	18 (4.1)	10 (1.4)	28 (2.4)	
Housewife	00 (0.0)	71 (10.0)	71 (6.2)	
Student	230 (52.6)	340 (47.6)	570 (49.5)	
Self employed	93 (21.3)	152 (21.3)	245 (21.3)	
Employed	44 (10.0)	56 (7.8)	100 (8.7)	
Monthly income				<0.0001
< 50,000FCFA	262 (61.1)	450 (66.3)	712 (64.3)	
50–100,000FCFA	92 (21.4)	172 (25.3)	264 (23.8)	
>100,000FCFA	75 (17.4)	57 (8.3)	132 (11.9)	
Family history of CVD				0.337
Yes	145 (32.8)	227 (31.8)	370 (32.2)	
No	296 (67.2)	486 (68.2)	782 (67.8)	
Overall BMI, mean ± SD (kg/m^2^)	24.4 ± 3.9	25.6 ± 5.0	25.2 ± 4.7	<0.0001
Normal (18.5 ≤ BMI ≤ 24.9)	280 (64.4)	348 (49.4)	628 (55.1)	
Overweight (25 ≤ BMI ≤ 29.9)	133 (30.6)	263 (37.4)	396 (34.8)	
Obese (≥30 kg/m^2^)	22 (5.1)	93 (13.2)	115 (10.1)	
Perception of weight				0.004
Underweight	13 (2.9)	32 (4.5)	45 (4.0)	
Normal	392 (90.9)	583 (82.8)	975 (85.9)	
Overweight	25 (5.9)	84 (11.9)	109 (9.5)	
Obese	1 (0.3)	5 (0.8)	6 (0.6)	
Perception of lifestyle				0.536
Very stressful	22 (4.9)	36 (5.2)	58 (5.0)	
Stressful	115 (25.9)	157 (22.0)	272 (23.6)	
Relatively stressful	218 (49.3)	354 (49.9)	572 (49.6)	
Free from stress	88 (19.9)	163 (22.9)	251 (21.8)	
Smoking status				<0.0001
Current	60 (13.6)	13 (1.9)	73 (6.4)	
Former	53 (12.1)	08 (1.1)	61 (5.4)	
Never	325 (74.2)	688 (97.0)	1013 (88.2)	
Alcohol Use				<0.0001
Yes	158 (35.7)	96 (13.4)	254 (21.9)	
No	284 (64.3)	619 (86.6)	903 (78.1)	
Frequency of eating healthy food				0.539
Not everyday	366 (83.0)	602 (84.3)	968 (83.3)	
Everyday	75 (17.0)	112 (15.7)	187 (16.7)	
Number of times you do at least 30mins of physical activity in a week				0.001
0–2 times	168 (42.1)	363 (54.1)	531 (49.6)	
3 times or more	231 (57.9)	308 (45.9)	539 (50.4)	

Numbers represent frequency and percentage in brackets for categorical variables and means ± standard deviation for continuous variables. Group means compared using independent t-test; proportions compared using chi square test

*Abbreviations*: *CVD* cardiovascular disease, *BMI* body mass index, *SD* standard deviation, *FCFA* Central African francs

### Cardiovascular disease types, warning features and risk factors awareness

Overall knowledge on CVD was poor with mean score of 12.1 on a total of 25 points similar across sex. However some peculiarities were seen in the CVD knowledge components explored (Tables 2 and 3).

**Table 2:** Knowledge about CVD types, their risk factors and symptoms of heart attack and stroke according to sex among adults in Buea, Cameroon, 2016

	Male	Female	Total	*p*-value
Types of Cardiovascular disease				
Coronary heart disease				0.011
Yes	177 (40.0)	255 (35.9)	432 (37.5)	
I don’t know	254 (57.5)	450 (63.4)	704 (61.1)	
No	11 (2.5)	05 (0.7)	16 (1.4)	
Stroke				0.010
Yes	86 (19.7)	102 (14.6)	188 (16.6)	
I don’t know	288 (66.1)	520 (64.4)	808 (71.2)	
No	62 (14.2)	77 (11.0)	139 (12.2)	
Peripheral arterial disease				0.001
Yes	111 (25.2)	130 (18.5)	241 (21.0)	
I don’t know	292 (66.2)	536 (76.1)	828 (72.3)	
No	38 (8.6)	38 (5.4)	76 (6.6)	
Rheumatic heart disease				0.027
Yes	115 (26.2)	143 (20.3)	258 (22.6)	
I don’t know	293 (66.7)	522 (74.1)	815 (71.3)	
No	31 (7.1)	39 (5.5)	70 (6.1)	
Congenital heart disease				0.028
Yes	131 (29.7)	171 (24.2)	302 (26.3)	
I don’t know	281 (63.7)	503 (71.1)	784 (68.3)	
No	29 (6.6)	33 (4.7)	62 (5.4)	
Deep vein thrombosis & Pulmonary embolism				0.003
Yes	120 (27.3)	145 (20.6)	265 (23.1)	
I don’t know	293 (66.6)	534 (75.7)	827 (72.2)	
No	27 (6.1)	26 (3.7)	53 (4.6)	
Risk factors for Cardiovascular disease				
Family history of cardiovascular disease				0.537
Yes	215 (49.0)	329 (46.8)	544 (47.6)	
I don’t know	150 (34.2)	263 (37.4)	413 (36.2)	
No	74 (16.9)	111 (15.8)	185 (16.2)	
Smoking				0.730
Yes	359 (81.8)	573 (82.1)	932 (82.0)	
I don’t know	61 (13.9)	101 (14.5)	162 (14.2)	
No	19 (4.3)	24 (3.4)	43 (3.8)	
Unhealthy diet				0.535
Yes	305 (69.5)	500 (71.2)	805 (70.6)	
I don’t know	99 (22.6)	158 (22.5)	257 (22.5)	
No	35 (8.0)	44 (6.3)	79 (6.9)	
Lack of exercise				0.175
Yes	297 (68.0)	466 (66.4)	763 (67.0)	
I don’t know	91 (20.8)	174 (24.8)	265 (23.3)	
No	49 (11.2)	62 (8.8)	111 (9.7)	
Obesity				0.269
Yes	294 (67.1)	500 (71.3)	794 (69.7)	
I don’t know	113 (25.8)	163 (23.3)	272 (24.2)	
No	31 (7.1)	38 (5.4)	69 (6.1)	
Stress				0.211
Yes	322 (73.5)	512 (72.8)	834 (73.1)	
I don’t know	93 (21.6)	136 (19.3)	229 (20.1)	
No	23 (5.3)	55 (7.8)	78 (6.8)	
High levels of LDL cholesterol				0.053
Yes	158 (36.0)	223 (31.8)	381 (33.4)	
I don’t know	251 (57.2)	447 (63.7)	698 (61.2)	
No	30 (6.8)	32 (4.6)	62 (5.4)	
High blood pressure				0.048
Yes	306 (69.5)	532 (75.7)	838 (73.3)	
I don’t know	110 (25.0)	147 (20.9)	257 (22.5)	
No	24 (5.5)	24 (3.4)	48 (4.2)	
Diabetes mellitus				0.425
Yes	260 (59.1)	433 (61.9)	693 (60.8)	
I don’ know	129 (29.3)	203 (29.0)	332 (29.1)	
No	51 (11.6)	64 (9.1)	115 (10.1)	
Symptoms of Heart attack				
	**Men**	**Women**	**Total**	***p*** **-value**
Pain in the neck, jaw or back				0.212
Yes	116 (26.5)	213 (30.5)	329 (28.9)	
I don’t know	222 (50.7)	351 (50.2)	573 (50.4)	
No	100 (22.8)	135 (19.3)	235 (20.7)	
Feeling weak, light-headed or faint				0.080
Yes	216 (49.1)	379 (53.8)	595 (52.0)	
I don’t know	170 (38.6)	266 (37.7)	436 (38.1)	
No	54 (12.3)	60 (8.5)	114 (10.0)	
Chest pain or discomfort				0.404
Yes	253 (57.4)	401 (57.0)	654 (57.2)	
I don’t know	152,934.5)	258 (36.7)	410 (35.8)	
No	36 (8.2)	44 (6.3)	80 (7.0)	
Pain or discomfort in arms or shoulder				0.112
Yes	114 (26.0)	147 (21.2)	261 (23.0)	
I don’t know	232 (52.8)	407 (58.6)	639 (56.3)	
No	93 (21.2)	141 (20.3)	234 (20.6)	
Shortness of breath				0.038
Yes	285 (64.5)	504 (70.9)	789 (68.4)	
I don’t know	134 (30.3)	185 (26.0)	319 (27.7)	
No	23 (5.2)	22 (3.1)	45 (3.9)	
Symptoms of Stroke				
Sudden numbness/weakness in face, arm or leg				<0.0001
Yes	303 (69.5)	563 (80.0)	866 (76.0)	
I don’t know	109 (25.0)	116 (16.5)	225 (19.7)	
No	24 (5.5)	25 (3.6)	49 (4.3)	
Sudden confusion, trouble speaking or understanding others				0.386
Yes	273 (62.8)	437 (62.5)	710 (62.6)	
I don’t know	122 (28.0)	181 (25.9)	303 (26.7)	
No	40 (9.2)	81 (11.6)	121 (10.7)	
Sudden poor vision in one or both eyes				0.923
Yes	155 (35.8)	258 (37.0)	413 (36.5)	
I don’t know	197 (45.5)	311 (44.6)	508 (44.9)	
No	81 (18.7)	129 (18.5)	210 (18.6)	
Sudden dizziness, difficulty walking or loss of balance				0.142
Yes	295 (67.4)	510 (72.8)	805 (70.7)	
I don’t know	118 (26.9)	155 (22.1)	273 (24.0)	
No	25 (5.7)	36 (5.1)	61 (5.4)	
Severe headache with no known cause				0.022
Yes	110 (25.3)	228 (32.7)	338 (29.8)	
I don’t know	222 (51.0)	308 (44.1)	530 (46.8)	
No	103 (23.7)	162 (23.2)	265 (23.4)	

Numbers represent frequency and percentage in brackets for categorical variables

*Abbreviations*: *LDL* low density lipoprotein, group comparisons done with chi square test

**Table 3:** Comparison of population mean knowledge scores about CVD types, risk factors, symptoms of heart attack and stroke, and overall CVD knowledge according to sex in Buea, Cameroon, 2016

	Male (*n* = 443) mean ± SD	Female (*n* = 716) mean ± SD	Total (1159) mean ± SD	*P*-value
Knowledge of CVD types (total score = 6)	1.65 ± 2.11	1.31 ± 1.89	1.44 ± 1.98	0.004
Knowledge of CVD risk factors (total score = 9)	5.68 ± 2.64	5.68 ± 2.61	5.68 ± 2.63	>0.999
Knowledge of heart attack symptoms (total score = 5)	2.20 ± 1.62	2.29 ± 1.60	2.26 ± 1.61	0.370
Knowledge of stroke symptoms (total score = 5)	2.55 ± 1.66	2.78 ± 1.59	2.69 ± 1.63	0.024
Overall CVD knowledge score (Total = 25)	12.08 ± 5.91	12.05 ± 5.42	12.07 ± 5.62	0.932

*Abbreviations*: *CVD* cardiovascular disease, *SD* standard deviation, group comparison of means done with independent samples t-test

Concerning types of cardiovascular disease, overall just 37.5, 16.6, 21, 22.6, 26.3 and 23.1% knew that coronary heart disease, stroke, peripheral arterial disease (PAD), rheumatic heart disease, congenital heart disease and deep vein thrombosis (DVT)/pulmonary embolism (PE) respectively, were various types of CVD. The mean score of knowledge on CVD types was 1.4 on a total of 6 points and was better in men compared to women (*p* = 0.004).

For CVD risk factors, participants were aware that smoking (82%), unhealthy diet (70.6%), lack of exercise (67.0%), obesity (69.7%), stress (73.1%), high blood pressure (HBP) (73.3%) and diabetes (60.8%) were potential risk factors for CVD but for family history of CVD, where up to 52.4% either were unaware it was a risk factor for CVD or thought it was not related to CVD. Women seemed more conscious that HBP was a risk factor for CVD (75.7% vs. 69.5%; *p* = 0.048). The mean score of knowledge on CVD risk factors was 5.7 on a total of 9 points, with no sex difference (*p* > 0.05).

With respect to symptoms of heart attack and stroke; most (68.4%) could identify shortness of breath as a symptom of heart attack, with women more aware than men (70.9% vs. 64.5%, *p* = 0.038). Participants were barely aware of other symptoms of heart attack such as pain in the left arm or shoulder (23.0%), pain in the jaw (28.9%), and weakness or light-headedness (52.0%). For stroke, most (76.0%) correctly identified sudden numbness/weakness in the face, arm or leg as a symptom, with women having better knowledge than men (80.0% vs. 69.5%, *p* < 0.0001). Quite few (29.8%) were aware that a sudden severe headache with no known cause could be a feature of stroke. This limited knowledge was observed more in the men (25.3% vs. 29.8%, *p* = 0.022).

### Factors associated with overall good knowledge on cardiovascular disease

In multivariable logistic regression analysis (see Table 4), high level of education [(aO.R. = 2.26 (1.69–1.41), *p* < 0.0001], monthly income greater than 100,000FCFA (~USD 200) [aO.R. = 1.64 (1.07–2.51), *p* = 0.023], family history of CVD [aO.R. = 1.59 (1.21–2.09), *p* = 0.001] and being a former smoker [1.11 (1.02–1.95), *p* = 0.043] were independently associated with overall good knowledge on CVD.

**Table 4:** Factors associated with overall good knowledge for cardiovascular disease among adults in Buea, South-west region, Cameroon, 2016

Characteristics	Bivariate analysis		Multivariable analysis	
	O.R. (95% CI)	*p*-value	aO.R. (95% CI)	*p*-value
Sex				
Male	0.92 (0.72–1.16)	0.469	0.81 (0.60–1.11)	0.238
Female	Ref		Ref	
Age categories				
≤27 years	1.26 (1.01–1.59)	0.044	1.05 (0.78–1.41)	0.774
>27 years	Ref		Ref	
Level of education				
Low-moderate (secondary + high school)	Ref		Ref	
High (diploma + university)	1.87 (1.48–2.37)	<0.0001	2.26 (1.69–3.02)	<0.0001
Monthly income				
<50,000FCFA	Ref		Ref	
50–100,000FCFA	0.98 (0.74–1.30)	0.886	1.09 (0.80–1.48)	0.582
>100,000FCFA	1.39 (1.01–2.02)	0.048	1.64 (1.07–2.51)	0.023
Frequency of physical activity (30mins at least)				
0–2 times/ week	Ref		Ref	
≥3 times/ week	1.25 (0.98–1.59)	0.071	1.19 (0.93–1.54)	0.171
Eating healthy food				
Not everyday	Ref		-	-
Everyday	1.67 (0.47–5.97)	0.427	-	-
Family history of CVD				
No	Ref		Ref	
Yes	1.32 (1.03–1.69)	0.029	1.59 (1.21–2.09)	0.001
Alcohol use				
Yes	Ref		Ref	
No	1.38 (1.02–1.93)	0.031	1.26 (0.85–1.86)	0.250
Smoking status				
Current	Ref		Ref	
Former	1.10 (1.01–1.90)	0.031	1.11 (1.02–1.95)	0.043
Never	1.24 (1.11–2.01)	0.049	1.12 (0.58–2.17)	0.738

*Abbreviations*: *O.R.* odds ratio, *aO.R.* adjusted odds ratio, *CI* confidence interval, *Ref* reference category

## Discussion

In this large community-based study from Buea in the southwest region of Cameroon, we have found that population knowledge regarding CVD is sub-optimal with over half (52.2%) of the participants having an overall poor knowledge score. While awareness on CVD risk factors was relatively good, the majority had poor knowledge regarding CVD types and warning signs of heart attack and stroke. A number of sex differences were observed with respect to knowledge on stroke and heart attack warning signs which was mostly better among women. Having a higher monthly income, high level of education, a family history of CVD and being a former smoker were associated with increased likelihood of having moderate-to-good knowledge on CVDs. These findings have implications for policy makers in their considerations of strategies in the fight against NCDs and CVD in particular.

The overall low level of knowledge on CVD observed in our study is similar to findings among primary care physicians in the West region of Cameroon [17] and among university staff in Nigeria [19]. Similar worrisome knowledge levels have been obtained across other settings [10, 12]. While participants had a moderate-to-good knowledge on risk factors, this paradoxically occurred in the context of reported unhealthy diets, and/or lifestyles, potentially increasing populations’ risk for CVDs. Further investigation of drivers for poor health behaviours is thus warranted. They fell short in identifying various types of CVD and warning signs of heart attacks which led to an overall low CVD knowledge in the population. We observed a tendency to overweight (mean BMI of 25.2 kg/m^2^) in our study population, albeit the majority perceived their weight as being ‘normal’. Studies have shown that the heavier people become, the earlier in life they develop CVDs and their complications, resulting in reduced quality of life and life expectancy [29]. Further to this, only about 1 out of 7 individuals reported eating healthily (diet rich in fruits and vegetables and low in salt and saturated fats). This is less than the >50% reported by Awad et al. [25]. Almost half of our study participants were physically inactive, a finding which is alarming and higher than the 16.9% reported in West Cameroon [17], and almost twice as high as previous estimates for SSA [5]. Over one in five participants reported alcohol use. This was lower than observed in a population based study by Kengne et al. [30] and among physicians in Cameroon [17]. In a like manner, smoking was seen in 11% of participants, which is less than observed previously [30]. While this may seem interesting, it should be noted that this was based on self-report and hence, the actual use of these substances might have been under-estimated as they are somewhat considered to be ‘social ills’ in this setting. Also, other forms of tobacco use locally (like use of ‘snuff’) were not evaluated and hence could have contributed to this low rate.

With respect to CVD risk factors, participants had an overall good mean knowledge score, which was better in women compared to men. About two-thirds of the population could identify smoking, unhealthy diet (low in fruits, vegetables and high in salt and saturated fats), stress, high blood pressure, obesity and lack of exercise as potential risk factors for CVD. Similarly, participants in a Ugandan study identified stress and hypertension as common risk factors for CVD [31] and over two-thirds of adults in Nigeria identified hypertension as common risk factor for stroke [32].

Amidst the low awareness regarding warning features of heart attack, the commonest features identified were chest pain and shortness of breath. Awareness rates on most other symptoms fell below a quarter of the population. This was similar to a study among HIV patients in Kenya where the majority of participants (almost four in five) couldn’t identify warning signs for heart attack [33]. Mukattash and co-workers had similar observations in the majority of their respondents in Jordan [34].

For stroke, over two-thirds correctly identified sudden occurrence of weakness in face and limbs, or trouble speaking or difficulty walking as symptoms. This was in line with a study among university staff and students in Nigeria [19] and in Michigan [35], though it contrasts with the ‘blurred, double or loss of vision’ mostly identified among Australian urban dwellers [15]. That our study population was more aware of stroke compared to heart attack, could be consistent with the fear of handicap among the general population and also with the growing incidence of stroke in our setting [36].

We found in our study following multi-predictor analysis that; high (attainment of diploma and tertiary) level of education, being economically viable, having a family history of CVD, and being a former smoker were significant predictors of overall good knowledge on CVDs. As expected, participants with high levels of education were over twice as likely to have good knowledge on CVDs over their counterparts. There is evidence to support this observation [10, 37, 38] and could be explained by the fact that the more literate group as well as those with family history of CVD certainly had greater exposure to and could easily apprehend CVD health-related knowledge. Current smokers had poorer knowledge on CVDs and this corresponds to studies elsewhere; however this is at contrast with a Northern Irish study where no relationship was found between knowledge on CVD and smoking [38]. The heterogeneity observed across studies might in part be explained by the different study settings, methodologies and study populations.

To put this to context, a low awareness regarding warning features of heart attack and stroke was observed, with an overall suboptimal knowledge on CVD. These observations are occurring in a country with poor CVD risk profiles; around one in 4 individuals with raised blood pressure [39], about a third being overweight or obese [40], and increasing rates of stroke and stroke mortality [36] in Cameroon.

### Implications for policy and research

All-encompassing, our study findings have significant implications for health policy and further research. There is need for health authorities to design strategies and programs to improve community knowledge in the recognition of heart attacks with strokes. This would prompt individuals to seek urgent appropriate care, considering the value of time in the management of these ‘hypoxic-ischaemic’ conditions in averting the ensuing and sometimes irreversible sequelae. Amongst others, implementing and/or intensifying public health promotion with mass media campaigns via television, radio, and bill board advertisements, including text messages educating the public on warning signs of heart attacks, stroke and CVD risk factors are potential platforms to improve CVD knowledge and overall cardiovascular health of populations. Engaging community health workers in the implementation of these health promotion initiatives will potentially enhance their uptake. This is important especially for low socio-economic groups in whom we identified relatively sub-optimal knowledge, but who apparently carry the greatest disease burden. Some studies elsewhere have shown that such community awareness campaigns can be cost-effective and essential in reducing incidence and mortality from heart disease and strokes [41, 42]. Furthermore, considering the growing burden of NCD risk factors in children, early commencement of such awareness initiatives via school-based interventions (incorporating them into curricula) educating children on heart health and risk factors, proven to be beneficial elsewhere [16], are other agendas for consideration by the Cameroon ministry of public health. Creating enabling environments (such as promoting sales, availability and consumption of healthy foods like fruits and vegetables via subsidies, taxing tobacco, junk food and those high in salt, creating more pedestrian and cycling paths) are necessary combined efforts for sustainability of these interventions in primary CVD prevention. Put together, there is urgent need for funding to implement interventional and longitudinal studies to evaluate the impact of these strategies on future CVD burden. Importantly, these initiatives and strategies should be paralleled with regular monitoring and evaluation of their uptake, as well as examine trends in CVD, in a bid to continue identifying caveats needing attention.

Our study has a number of limitations. Its cross-sectional nature limited causality exploration. Some characteristics were based on self-report, with the risk of recall bias and consequent over- or under estimation. However, while CVD awareness alone is no guarantee for positive cardiovascular outcomes, good knowledge is clearly necessary for individuals to make informed decisions about their health by potentially adopting risk-free behaviours. Our findings are potentially generalizable to other parts of Cameroon and Africa, as the knowledge gap identified in our study is consistent with findings from West Cameroon [17] and also on par with sub-optimal awareness in a number of other African countries [19, 31–33]. However, it is possible that some towns in Cameroon, and to a broader extent, African countries, are at varied levels of urbanization and socioeconomic growth, with potential impact on knowledge/literacy levels. Those are important for consideration while interpreting our findings. Our study is a pioneer population based study in Cameroon that has explored and unearthed the level of CVD awareness as a whole and the gender differential. The large and rigorous probability sample gives strength to our estimates, as evidenced by narrow confidence intervals.

## Conclusions

We have found sub-optimal levels of knowledge regarding cardiovascular disease and warning features of CVD events (heart attack and stroke) in a population in south-west Cameroon. Having a high level of education, a high monthly income, family history of CVD and not smoking were associated with moderate-to-good knowledge. Community education on CVDs, targeting especially populations with low socio-economic status, may be beneficial in the combined efforts to achieve the reductions in heart attacks, strokes and other cardiovascular diseases formulated in the WHO Global Action Plan for the Prevention and Control of NCDs, 2013–2020.
